# Human methanogen diversity and incidence in healthy and diseased colonic groups using *mcrA *gene analysis

**DOI:** 10.1186/1471-2180-8-79

**Published:** 2008-05-20

**Authors:** Pauline D Scanlan, Fergus Shanahan, Julian R Marchesi

**Affiliations:** 1 Alimentary Pharmabiotic Centre, National University of Ireland, University College Cork, Cork, Ireland; 2 Department of Microbiology, National University of Ireland, University College Cork, Cork, Ireland; 3 Department of Medicine, National University of Ireland, University College Cork, Cork, Ireland; 4 School of Biosciences, Division of Microbiology, Cardiff University, Park Place, Cardiff, CF10 3US, Wales, UK

## Abstract

**Background:**

The incidence and diversity of human methanogens are insufficiently characterised in the gastrointestinal tract of both health and disease. A PCR and clone library methodology targeting the *mcrA *gene was adopted to facilitate the two-fold aim of surveying the relative incidence of methanogens in health and disease groups and also to provide an overview of methanogen diversity in the human gastrointestinal tract.

**Results:**

DNA faecal extracts (207 in total) from a group of healthy controls and five gastrointestinal disease groups were investigated. Colorectal cancer, polypectomised, irritable bowel syndrome and the control group had largely equivalent numbers of individuals positive for methanogens (range 45–50%). Methanogen incidence in the inflammatory bowel disease groups was reduced, 24% for ulcerative colitis and 30% for Crohn's disease. Four unique *mcrA *gene restriction fragment length polymorphism profiles were identified and bioinformatic analyses revealed that the majority of all sequences (94%) retrieved from libraries were 100% identical to *Methanobrevibacter smithii mcrA *gene. In addition, *mcrA *gene sequences most closely related to *Methanobrevibacter oralis *and members of the order *Methanosarcinales *were also recovered.

**Conclusion:**

The *mcrA *gene serves as a useful biomarker for methanogen detection in the human gut and the varying trends of methanogen incidence in the human gut could serve as important indicators of intestinal function. Although *Methanobrevibacter smithii *is the dominant methanogen in both the distal colon of individuals in health and disease, the diversity of methanogens is greater than previously reported. In conclusion, the low incidence of methanogens in Inflammatory Bowel Disease, the functionality of the methanogens and impact of methane production in addition to competitive interactions between methanogens and other microbial groups in the human gastrointestinal tract warrants further investigation.

## Background

The methanogens are a group of microorganisms within the Kingdom *Euryarchaeota *of the domain *Archaea *[1]. Methanogenesis is a process confined exclusively to the methanogens and utilises substrates such as hydrogen, CO_2_, acetate, formate, methanol and methylamines for methane generation. The methanogenic archaea are widely distributed in natural environments including the gastrointestinal tracts of ruminants, termites and humans [2-4]. Studies to date indicated that *Methanobrevibacter smithii *is the predominant archaeal species present in the human large intestine [2,5,6]. and so far only a second methanogenic species *Methanosphaera stadtmanae*, also a member of the order *Methanobacteriales *has been isolated from the human intestinal tract [7].

The reasons underlying methanogen incidence and their seemingly low diversity in human populations are of considerable interest to gastrointestinal microbial ecologists. An increasing interest in the methanogenic populations of the human gastrointestinal tract is also underpinned by their potential role as environmental factors in obesity [8] and pneumatosis cystoides intestinalis [9] and any possible association methanogens or methane production may have with gastrointestinal disease. As yet, no pathogenic *Archaea *have been characterised to date and the aetiological role, if any, methanogens play in disease is unknown [10,11]. Although no pathogenic archaeal species has been found [11], they are routinely identified in samples from the sites of oral infections [12-14] leading to speculation that they may be indirectly involved in disease processes by creating environmental conditions to facilitate the growth of microbes involved in pathogenesis.

In methane-producing humans methanogen numbers are approximately 1.6 × 10^8 ^to 8 × 10^9^/g faeces [15,16]. and in non-methanogenic humans 10^2 ^to 5 × 10^6^/g faeces [16]. Traditionally, the characterization of methanogens from the human intestinal environment has largely focused on phenotypic, microscopic, biochemical and nutritional classification methodologies [2,5,7]. and whilst these techniques are critical to gastrointestinal microbial studies they are time consuming and laborious [17]. Furthermore, molecular techniques for characterising microbial communities are considered more comprehensive and enable a more accurate overview of the diversity and functionality present in the human gastrointestinal tract [18,19].

This study has undertaken the optimisation of appropriate culture independent methodologies with the aim of investigating and characterising methanogen incidence and diversity in human faecal samples. A PCR and clone library methodology was employed using one set of previously published PCR primers [20] specific to the α subunit of methyl-coenzyme M reductase (*mcrA*), a functional gene that catalyzes the terminal step in the formation of methane by methanogens [21] and a second set of novel primers which were designed to specifically target the *mrtA *gene (*mcrA *gene holoenzyme) of *Methanosphaera stadtmanae*.

## Results and discussion

High molecular weight DNA, free from PCR inhibitors is critical to PCR analysis [22] and each DNA sample in this study was tested in order to ensure that a negative *mcrA *gene or *mrtA *gene PCR result were not as a consequence of poor DNA extraction and quality. An initial PCR targeting the 16S rRNA gene was used as a control measure for DNA quality and all faecal DNA extracts gave a positive signal for a 16S rRNA gene PCR assay. The *mcrA *gene was chosen as a proxy for methanogen detection as this gene can be readily amplified from *Mbb. smithii *(data not shown) which is the dominant methanogen in the human gut [2,5,6,23,24]. Furthermore, the detection limits of the primers for *mcrA *gene amplification were calculated and a positive PCR result obtained using the *mcrA *primer set from spiked samples at cell numbers ≥ 10^7 ^cells per gram of faeces using *Mbb. smithii *PS^T ^as the test organism. Therefore, results recorded and reported in this study were obtained from faecal samples where methanogen cell numbers equalled or exceeded 10^7 ^cells per gram of faeces making its detection level equivalent to that of the methane breath test [25] and real-time PCR assay using methanogen specific 16S rRNA gene primers [26].

Interestingly, the results from the PCR survey of methanogen frequency in different groups of individuals found that in the control, colorectal cancer, polypectomised and irritable bowel syndrome groups, the percentage of individual's positive for methanogens were largely equivalent and fell within a range of 45 – 50% (see Table 1). The percentage of individuals within the inflammatory bowel disease cohorts harboured methanogens at a lower rate of 30% for Crohn's disease (*P *< 0.1) and a significantly lower rate of 24% for Ulcerative Colitis compared to the control group (48%) (*P *< 0.01). Although these data can not be directly correlated with previous published studies, the same general trend was observed in a methane detection survey where methane excretion was detected in 54% of healthy controls, 53% of non gastrointestinal patients and 32% of gastrointestinal patients [27]. In that study the patients within the gastrointestinal disease group that had Inflammatory Bowel Disease had significantly lower methane detection; 13% for Crohn's Disease and 15% for Ulcerative Colitis [27]. Furthermore, methane production in the predominantly diarrhoeal conditions of Ulcerative Colitis and Crohn's Disease is reportedly almost non existent [28], a finding that may be due to loss of slow growing methanogens that are displaced during conditions of rapid gut transit. Collectively, these data for reduced methanogens in IBD may support the hypothesis that methane is not in fact biologically inert but may play a role in intestinal motility with consequences for irritable bowel syndrome associated constipation [29], diverticulitis [25] and encopresis [30].

**Table 1:** Overview of age and number of participants and the percentage of individuals positive for methanogens within each different disease group analysed.

Disease group	Number of individuals per group (n = 207 total)	Mean age, SD and range	Disease status	% Methanogen positive (*mcrA *gene)
Healthy Controls (HC)	44	25.8 ± 8.3 (range 19–56)	No history of gastrointestinal illness	48%
Crohn's Disease (CD)	27	41.3 ± 11.9 (range 25–70)	Both remission and relapse (CDAI >150 ^a^) individuals	30%
Ulcerative Colitis (UC)	29	49.0 ± 12.0 (range 32–70)	Both remission and relapse individuals ^b^	24% ^d^
Irritable Bowel Syndrome (IBS)	46	44.4 ± 12.8 yrs, (range 24–74)	Diagnosed according to the Rome II criteria ^c^	48%
Colorectal Cancer (CC)	31	60.3 ± 6.6 yrs, (range 45–70)	In remission, >95% receiving a wide range of medication	45%
Polypectomised (PP)	30	53.5 ± 9.1 yrs, (range 30–69)	Pre-cancerous polyps removed	50%

No data was available on the time of collection of samples and all samples analysed in this study were participants in larger trials. The individuals selected for each trial was done so in a randomised fashion with only the disease status of the host being the primary prerequisite for inclusion.

^a ^CDAI: Crohn's Disease Activity Index. Individuals with a CDAI greater than 150 are considered to be in relapse [47].

^b ^Diagnosed by clinician.

^c ^see [48].

^d ^Significantly (*P *< 0.01) different from Control group using Fischers' exact statistical test.

The results from the healthy group reported in this study are also similar to those obtained from a real time-PCR analysis of methanogen incidence and diversity in the human gastrointestinal tract using 16S rRNA gene as a target [31]. Only 12 adults were analysed as part of this study and 5/12 adults (42%) gave a positive PCR result. In the majority of samples analysed in the total study PCR amplicons generated were beyond the quantitative limits of the PCR assay. Furthermore, *Mbb. smithii *was the predominant sequence obtained from extensive sequence analysis with no *Msp*.*stadtmanae *sequences recovered [31].

**Figure 1:**
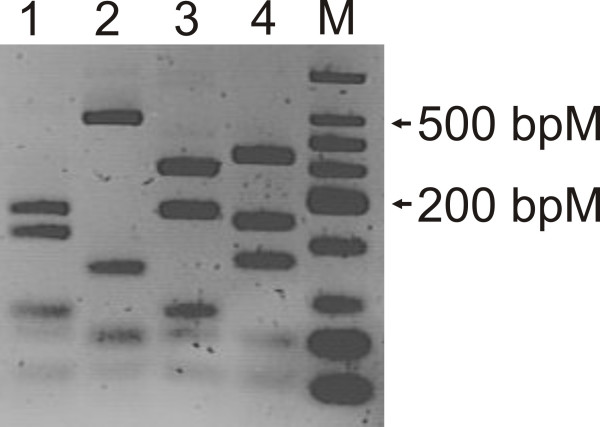
**RFLP analysis of *mcrA *gene amplicons**. 4% (w/v) agarose gel showing representatives of the four unique *mcrA *gene RFLP types identified in this study. Lane 1: RFLP profile of *mcrA *gene from *Mbb. smithii *PS^T ^(RFLP profile type A), Lane 2: RFLP profile identified in RFLP analysis of IBS clone library, sequence most closely related to *Mbb. oralis *and represented in Figure 2 by DC IBS-4, (RFLP profile Type B), Lane 3: RFLP profile generated from Ulcerative Colitis clone library analysis, most closely related to *Mbb. smithii *and represented in Figure 2 by clone DC UC-14, (RFLP profile Type C), Lane 4: RFLP profile Type D generated from Ulcerative Colitis clone library analysis and represented in Figure 2 by DC UC-6, uncultured methanogen clone, M: Low Weight Molecular DNA Ladder (Promega).

**Figure 2:**
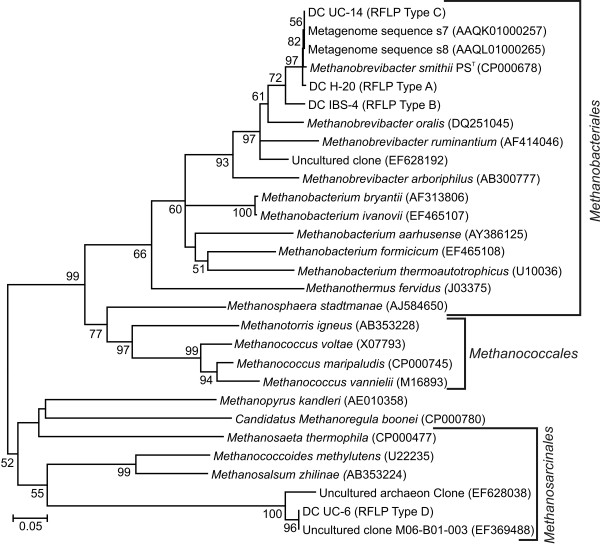
**Evolutionary relationships *mcrA *gene clone generated from this study to *mcrA *genes of cultured and uncultured methanogens**. The evolutionary history was inferred using the Neighbor-Joining method [49]. The bootstrap consensus tree inferred from 500 replicates [50] is taken to represent the evolutionary history of the taxa analyzed [50]. The percentage of replicate trees in which the associated taxa clustered together in the bootstrap test (500 replicates) are shown next to the branches [50] and any value below 50% was not shown. The tree is drawn to scale, with branch lengths in the same units as those of the evolutionary distances used to infer the phylogenetic tree. The evolutionary distances were computed using the Maximum Composite Likelihood method [51] and are in the units of the number of base substitutions per site. Codon positions included were 1^st^, 2^nd^, 3^rd ^and noncoding. All positions containing gaps and missing data were eliminated from the dataset (Complete deletion option). There were a total of 348 positions in the final dataset. The accession numbers are included in parenthesis after each entry.

With respect to the diversity of methanogens in the present study, restriction fragment length polymorphism (RFLP) analysis of the *mcrA *gene clones (558 clones in total) revealed only 4 unique RFLP types (designated RFLP Type A to D) the results of which are outlined in Table 2 and illustrated in Figures 1 and 2. In fact, all clone libraries generated with the exception of Ulcerative Colitis (UC), Crohn's Disease (CD) and Irritable Bowel Syndrome (IBS) exhibited a uniform RFLP profile identical to that obtained from *Mbb. smithii *PS^T ^and *Mbb. smithii *(DSM 2374) (designated Type A) with all clones that were subsequently sequenced showing 100% amino acid identity to *Mbb. smithii*. This finding reinforces the prevailing knowledge that *Mbb. smithii *is the dominant methanogen in the human gastrointestinal tract. Three additional RFLP profiles were identified in the UC, CD and IBS clone libraries. Amino acid sequence analysis of these clones indicated that RFLP type B from the IBS and Crohn's Disease clone library was most closely related to *Methanobrevibacter oralis*, Type C from Ulcerative colitis (UC-14) which, although exhibiting a unique RFLP profile, is most closely related to *Mbb. smithii *(see Figure 2) and a final RFLP type D (UC-6) which was identified in the Ulcerative Colitis library. Phylogenetic analysis indicated that UC-6's closet cultured relative was the alkaliphilic, halophilic and methylotrophic archaea *Methanosalsum zhilinae *[32] (see Figure 2 and supplementary Figure S1 for amino acid alignment of partial *mcrA *genes and identity matrix). The UC-6 sequence obtained in this study was also 100% identical to a sequence (accession number: EF369488) obtained from a human faecal sample from a unpublished study indicating that uncultured members of the methanogen outside the *Methanobacteriales *have been identified in other laboratories as well. The constructed phylogenetic tree further highlights these relationships amongst clone isolates from this study with cultured and uncultured methanogens (see Figure 2).

**Table 2:** Overview of *mcrA *clone library analysis

Sample	Number of Clones analysed (n = 558)	Number of different of RFLP profiles	Preliminary RFLP profile identification of methanogens
HC	224	1	*Mbb. smithii *– RFLP Type A
CC	190	1	*Mbb. smithii *– RFLP Type A
IBS	48	2	*Mbb. smithii *– RFLP Type A (19) and *Mbb. oralis*-Type B (29)
UC	48	3	*Mbb. smithii *– RFLP Type A (46), *Mbb. smithii *– RFLP Type C (1) and Unknown (related to *Methanosalsum zhilinae*) – RFLP Type D (1)
CD	48	2	*Mbb. smithii *– RFLP Type A (45) and *Mbb. oralis *– RFLPType B (3)

Several mismatches to the *mrtA *gene sequence of *Msp*.*stadtmanae *were evident with the *mcrA *primer set and phylogenetic analysis and tree construction illustrates that although the *mrtA *gene of *Msp. stadtmanae *encodes an enzyme of the same function and inhabits the same niche it is phylogenetically distinct from *mcrA *gene sequences of *Mbb. smithii *and related clones (see Figure 2). A second set of primers were designed and optimised for *mrtA *gene amplification serving as a marker for *Msp. stadtmanae *detection. PCR and clone library analysis indicate that the primers were highly specific for and readily amplified DNA from pure *Msp. stadtmanae *DNA at concentrations less than 0.01 ng ul^-1^. However, no positive PCR results were obtained for *mrtA *gene from faecal DNA extracts in this study. Further analysis of available 16S rRNA gene libraries from published literature [6,31] and analysis *in silico *of metagenomic clones libraries generated from human faecal samples also support the idea that *Msp. stadtmanae *is not a common feature of the human gastrointestinal microbiota. Therefore, it is quite possible that if *Msp. stadtmanae *was present in any of the faecal samples analysed in this study its concentration was below the detection levels of this novel primer set.

*Methanobrevibacter smithii *and *Msp*.*stadtmanae *are phylogenetically diverse methanogens and the metabolic capacity of both microbes may provide evidence for the predominance of *Mbb*.*smithii *in the human gastrointestinal tract. Genome comparisons between both methanogens indicate that *Mbb. smithii *is more adapted to the gut environment in terms of persistence, metabolic versatility and capacity for genomic evolution [33]. Not only is the *Mbb. smithii *genome significantly enriched for genes involved in CO_2_, H_2 _and formate utilisation during methanogenesis, *Mbb. smithii *also possesses the capacity for non methanogenic removal of other bacterial fermentation end-products, namely methanol and ethanol. Although both genomes exhibit limited global synteny, *Msp*.*stadtmanae *has the most restricted energy metabolism of any archaea studied to date [34] and can only use hydrogen to reduce methanol to methane. It is possible that this limited substrate range could be a factor in competition between *Msp*.*stadtmanae *and other microorganisms such as *Mbb. smithii *and the sulphate reducing bacteria which also require hydrogen for growth [35]. However, further insight into *Msp*.*stadtmanae *incidence and interaction is now possible with this novel primer set which also have application to the rumen gut and other ecosystems where *Msp*.*stadtmanae *like sequences have been reported [36,37]

The functionality of methanogens appears to be strongly associated and facilitated by the presence and activities of other microbial groups [38,39]. If indeed the carriage of methanogens is dependent upon or supported by other microbial groups and their activities, these differences observed between the methanogen frequency in certain disease and control groups compared to inflammatory bowel disease is quite possibly linked to the reduced bacterial diversity that is routinely reported in this gastrointestinal disease conditions [40-42] Considering these points, it is reasonable to suggest the presence of methanogens in human gastrointestinal tract is part due to the presence of other microbial group or groups with fermentation capacities that generate sufficient H_2 _and substrates to support methanogenesis in a competitive environment. The presence of such a bacterial population could be present due to genetic host factors, diet, intestinal transit time, stochastic and other environmental factors that would support such an overall symbiotic community. As such the reduced frequency of methanogens evident within the IBD groups could possibly serve as a biomarker of altered microbial diversity and metabolic processes within the human gastrointestinal tract.

## Conclusion

This exploration of a functionally significant microbial group provides a comprehensive survey of methanogen incidence and diversity in both health and disease of the human gastrointestinal tract using a culture independent approach. The limited diversity of methanogens as evidenced by the *Mbb. smithii *dominance of clone libraries and failure to amplify *Msp. stadtmanae *from faecal samples raises interesting ecological questions on the nature of microbial competition for resources in the human gut. Furthermore, the identification of a *mcrA *gene sequences only distantly related to cultured methanogens is of significant interest and highlights the necessity of a culture independent approach when surveying diversity of this fastidious group of microorganisms and also the requirement to better our cultivation techniques to gain access to novel and uncultured microbes. In addition, the results of this study have highlighted some important points on the nature of methanogen carriage and their prospect as markers for altered gastrointestinal function. The reduced incidence of hydrogen utilising microbes in IBD could also serve as a biomarker for altered biochemical activities in the intestinal tract of certain individuals and warrants further exploration. It is now possible with the methods outlined in this study that functional analysis of *mcrA *gene as a proxy for methane production using RNA as a template could have relevance for the further study of clinical conditions related to methanogen carriage and methane production and also the competitive interactions between methanogens and other microbial groups.

## Methods

### Sample collection and total DNA extraction

A total of 207 faecal samples from individuals within diseased and healthy colonic groups were obtained and faecal samples were stored at -80°C until analyses (see Table 1 for an overview of participants). All samples were stored and processed in the same manner. Samples were thawed on ice and approximately 220 mg of stool was used for DNA extraction using the Qiagen MiniStool kit (Qiagen, Hilden, Germany) as per manufacturer's instructions for pathogen isolation, with an initial bead-beating step of 30 s and heating step of 85°C. DNA concentration was determined using the Nanodrop spectrophotometer. The details of each group are outlined in Table 1. No individuals were receiving or had received antibiotics within three months of faecal sample collection.

### PCR amplification of 16S rRNA as DNA quality control

Primers 27f and 1492r specific for the bacterial 16S rRNA were used in PCR mixtures containing 50 μl containing 1 X TAE Buffer (20 mM Tris-HCl pH 8.4, 50 mM KCl), 3 mM MgCl_2, _200 μM of each deoxynucleoside triphosphate, 1.25 U of *Taq *polymerase (Invitrogen), 10 pmol of each primer and 5 ng of extracted DNA. PCR was conducted in duplicate in a MJ Research PTC-200 Thermal Cycler and cycling conditions were as follows: 94°C for 5 min initial denaturation, followed by 30 cycles of 94°C for 30 s, 55°C for 40 s and 72°C for 1 min 30 s, with a final extension of 72°C for 10 min. PCR amplicons were analysed by electrophoresis in 1% w/v agarose containing ethidium bromide (0.25 mg ml^-1^) and 1 X TAE Buffer [43] with an applied voltage of 5 V cm^-1^. DNA was visualised by UV illumination (302 nm).

### PCR procedure for *mcrA *gene amplification

Previously published primers ME1 (5'-GCMATGCARATHGGWATGTC-3') and ME2 (5'-TCATKGCRTAGTTDGGRTAGT-3') [20] specific for *mcrA *gene were employed. Both cycling and reagent concentrations for PCR reactions targeting the *mcrA *gene were according to the methodology of Hales *et al*., [20]. Genomic DNA from *Methanocaldococcus infernus *ME^T^, *Mbb. smithii *(DSM 2374) and *Mbb. smithii *PS^T ^were used as positive PCR controls. PCR amplicons were analysed as previously outlined for 16S rRNA gene PCR and target sequences for *mcrA *gene were approximately 760 bp.

### Primer design and PCR procedure for *mrtA *amplification

Analysis *in silico *of *mcrA *gene primers and the *mrtA *gene sequence of *Msp. stadtmanae *alignments indicated that several nucleotide mismatches were evident between the sequences. A second set of primers for the specific amplification of the *mrtA *gene from *Msp. stadtmanae *MCB-3^T ^were designed. Reference *mcrA *gene sequences and the *mrtA *gene from *Msp. stadtmanae *MCB-3^T ^were imported into Bioedit from Genbank. Nucleotide sequences were aligned using CLUSTALW and primers were designed to target two unique sites specific to *mrtA *gene from *Msp. stadtmanae *and are as follows; MrtA_for (5' AAA CAA TCA ACC ACG CAC TC 3') and MrtA_rev (5' GTG AGC CCA ATC GAA GGA 3'). Initial PCR procedure was tested and optimised using a gradient MJ Research PTC-200 Thermal Cycler on genomic DNA from *Msp. stadtmanae *MCB-3^T^. The primers were also tested on DNA from *Methanocaldococcus infernus *ME^T^, *Mbb. smithii *(DSM 2374) and *Mbb. smithii *PS^T^. PCR amplicons generated from *Msp. stadtmanae *MCB-3^T ^were also cloned out (using the procedure for *mcrA *gene cloning) to further assess the specificity of the primer set. Final PCR mixtures for the amplification for the *mrtA *gene from faecal DNA extractions consisted of 50 μl containing 20 mM Tris-HCl (pH 8.4), 50 mM KCl, 3 mM MgCl_2, _50 mM concentrations of each dNTP, 1.25 U of *Taq *polymerase (Invitrogen), 10 pmol of each primer, and 10 ng of genomic DNA. PCRs were conducted in duplicate in a MJ Research PTC-200 Thermal Cycler and cycling conditions were as follows: 95°C for 5 min initial denaturation, followed by 35 cycles of 94°C for 40 s, 55°C for 40 s and 72°C for 90 s with a final extension of 72°C for 5 min. All PCR reactions were repeated on a separate occasion to verify results. Target sequences were approximately 1170 bp.

### Determination of *mcrA *gene PCR primer detection limits

Faecal samples that tested negative for *mcrA *gene products as using PCR protocol as previously outlined were used to conduct spiking experiments in order to determine the limits of detection for each primer set. Liquid pure culture *Mbb. smithii *(DSM 2374) was obtained from the DSMZ and the numbers of cells per ml were determined using a haemocytometer according to standard procedure. Known number of cells were added to negative faecal samples at concentrations of 10^9^, 10^8^, 10^7^, 10^6 ^and 10^5 ^cells per g of faecal sample and extracted as previously outlined. A non spiked faecal sample and a pure cell culture of each microbial group were included as controls. All experiments were conducted in duplicate and PCR for *mcrA *gene amplification was conduced on each extract as previously outlined. DNA from *Methanocaldococcus infernus *ME^T ^and *Mbb. smithii *(DSM 2374) were included as PCR controls in each experiment.

### Statistical analysis of methanogen detection in different groups

The results from the *mcrA *gene PCR were tabulated and the numbers of individuals positive for methanogens are expressed as a percentage of the total number of individuals analysed within each specific group. Fisher's Exact test was used to analyse the statistical significance of the frequency of methanogen carriage from each different disease cohorts compared to the healthy control group.

### Cloning of *mcrA *gene and analysis of the methanogen diversity

Four randomly selected individuals per group that gave a positive PCR result for *mcrA *gene amplification were chosen to generate *mcrA *gene clone libraries. Four PCR products for each group were pooled and cleaned (using the Qiagen Gel Extraction Kit), before cloning using TOPO^® ^XL PCR Cloning Kit (Invitrogen). Clones were grown overnight on LB agar (1.5% w/v agar) containing kanamycin for selection (50 μg ml^-1^). Kanamycin resistant colonies were picked and colony PCR was performed. Briefly, the picked colony was suspended in 20 μl of sterile distilled H_2_O and heated to 95°C for 5 min before adding 1 μl aliquot to each respective PCR mixture as previously described. Products were checked for the correct size insert on 1% (w/v) agarose gel as previously described. Restriction fragment length polymorphism (RFLP) using the enzymes *Alu *I and *Mse *I of cloned *mcrA *PCR products and *mcrA *gene PCR product of *Mbb. smithii *(DSM 2374) and *Mbb. smithii *PS^T ^was conducted. RFLP products were electrophoresed for 3 hours at constant 100 V through 2% (w/v) agarose and stained using ethidium bromide.

### Phylogeny of *mcrA *gene clones

Nucleotide sequences for *mcrA *were analysed using the tBLASTx (translated query versus translated database) function of Basic Local Alignment Search Tool (BLAST) [44]. Phylogenetic trees were constructed using relevant *mcrA *gene sequences in order to highlight phylogenetic relationships between the sequences retrieved in this study and other methanogens. Translated nucleotide sequences for McrA were edited and aligned with relevant sequences obtained from GenBank using the CLUSTALW [45] function of MEGA 4 [46]. Unambiguously aligned sequence regions were used to construct bootstrap-supported (500 resamplings) phylogenetic trees.

### Nucleotide sequence accession numbers

Nucleotide sequence numbers have been deposited in the EMBL Nucleotide Sequence Database under accession no. EMBL: AM921680, EMBL: AM921681, EMBL: AM921683 and EMBL:AM921684.

## Authors' contributions

PDS designed and performed all laboratory experiments including bioinformatic analysis, and drafted the manuscript, JRM helped in the experimental and primer design, bioinformatic analysis and the writing of the manuscript. FS helped in the writing of the manuscript. All authors read and approved the final manuscript.
